# Host Cationic Antimicrobial Molecules Inhibit S. aureus Exotoxin Production

**DOI:** 10.1128/msphere.00576-22

**Published:** 2023-01-04

**Authors:** Patrick M. Schlievert, Samuel H. Kilgore, Lisa A. Beck, Takeshi Yoshida, Aloysius J. Klingelhutz, Donald Y. M. Leung

**Affiliations:** a Department of Microbiology and Immunology, Carver College of Medicine, University of Iowa, Iowa City, Iowa, USA; b Department of Dermatology, University of Rochester Medical Center, Rochester, New York, USA; c Department of Pediatrics, National Jewish Health, Denver, Colorado, USA; University of Kentucky

**Keywords:** *Staphylococcus aureus*, calprotectin, cytotoxins, defensins, lysostaphin, lysozyme, superantigens

## Abstract

Innate immune molecules, including antimicrobial peptides (for example, defensins) and lysozyme, function to delay or prevent bacterial infections. These molecules are commonly found on mucosal and skin surfaces. Staphylococcus aureus is a common pathogen and causes millions of infections annually. It is well known that innate immune molecules, such as defensins and lysozyme, either poorly inhibit or do not inhibit the growth of S. aureus. Our current studies show that the α-defensin human neutrophil α-defensin-1 (HNP-1) and lysozyme inhibit exotoxin production, both hemolysins and superantigens, which are required for S. aureus infection. HNP-1 inhibited exotoxin production at concentrations as low as 0.001 μg/mL. Lysozyme inhibited exotoxin production at 0.05 to 0.5 μg/mL. Both HNP-1 and lysozyme functioned through at least one two-component system (SrrA/B). The β-defensin human β-defensin 1 (HBD-1) inhibited hemolysin but not superantigen production. The cation chelator S100A8/A9 (calprotectin), compared to EDTA, was tested for the ability to inhibit exotoxin production. EDTA at high concentrations inhibited exotoxin production; these were the same concentrations that interfered with staphylococcal growth. S100A8/A9 at the highest concentration tested (10 μg/mL) had no effect on S. aureus growth but enhanced exotoxin production. Lower concentrations had no effect on growth or exotoxin production. Lysostaphin is regularly used to lyse S. aureus. The lytic concentrations of lysostaphin were the only concentrations that also inhibited growth and exotoxin production. Our studies demonstrate that a major activity of innate defensin peptides and lysozyme is inhibition of staphylococcal exotoxin production but not inhibition of growth.

**IMPORTANCE** Staphylococcus aureus causes large numbers of both relatively benign and serious human infections, which are mediated in large part by the organisms’ secreted exotoxins. Since 1921, it has been known that lysozyme and, as shown later in the 1900s, other innate immune peptides, including human neutrophil α-defensin-1 (HNP-1) and human β-defensin 1 (HBD-1), are either not antistaphylococcal or are only weakly inhibitory to growth. Our study confirms those findings but, importantly, shows that at subgrowth inhibitory concentrations, these positively charged innate immune peptides inhibit exotoxin production, including both hemolysins and the superantigen toxic shock syndrome toxin-1. The data show that the principal activity of innate immune peptides in the host is likely to be inhibition of exotoxin production required for staphylococcal mucosal or skin colonization rather than growth inhibition.

## INTRODUCTION

Staphylococcus aureus causes a significant number of both relatively benign as well as very serious human infections that are mediated in large part by secreted virulence factors, including exotoxins (1–4). Our prior studies using the protein synthesis inhibitor clindamycin (5) and signal transduction inhibitor glycerol monolaurate (6) suggest that their abilities to inhibit S. aureus exotoxin production are distinct from their abilities to inhibit S. aureus growth. The underlying mechanism for clindamycin inhibition of exotoxin production independent of growth remains unknown. The ability of glycerol monolaurate to inhibit exotoxin production resides in its ability to dissipate the potential difference across the bacterial plasma membranes, similar to reutericyclin, which is produced by some lactobacilli (6, 7).

Positively charged hemoglobin peptides (8) and positively charged chitosan (9) also inhibit exotoxin production at concentrations that do not affect S. aureus growth. Many menaquinone analogues also share exotoxin synthesis inhibition abilities at concentrations that do not affect growth (10, 11). These inhibitory molecules exert part of their activities through inhibition of two-component system (TCS) signaling, including through the global TCS staphylococcal respiratory response A/B (SrrA/B) (10, 11). The molecular mechanism of action of SrrA/B remains unknown, making it difficult to know how these antimicrobial molecules interact with the two-component system. It is known that SrrA/B is a repressor of exotoxin production under anaerobic conditions, and it is likely that SrrA/B is sensing the redox potential in the plasma membrane (12, 13); the system is derepressed in the presence of ≥2% oxygen (13, 14). It has been impossible to find conditions for S. aureus to produce exotoxins under anaerobic conditions despite many attempts, suggesting that SrrA/B may be the top regulator of exotoxin production by S. aureus (5, 14). For example, SrrA/B is known to regulate the well-known global accessory gene regulator system, which itself also regulates the production of many exotoxins (13).

The separable growth and exotoxin synthesis-inhibitory activities are expected to be of value in the development of novel anti-infective agents. For example, it is desirable to interfere with toxic shock syndrome toxin-1 (TSST-1) synthesis vaginally to prevent menstrual TSS while not interfering with the normal microbiome, per the Food and Drug Administration (15). Additionally, the separation of activities may define more clearly how the human host responds with various antimicrobial peptides to inhibit staphylococcal exotoxin production and in this way prevent staphylococcal colonization and consequent overt infections.

We undertook studies to explore the exotoxin synthesis inhibition activities of human defensin peptides, lysozyme, the metal chelators S100A8/9 peptides and EDTA, lysostaphin, and α-lactalbumin (due to structural similarity with lysozyme).

## RESULTS AND DISCUSSION

Human neutrophil α-defensin-1 (HNP-1) was tested for its ability to inhibit growth and exotoxin production by S. aureus MNPE, MN8, and MNPA. All three of these strains were isolated originally from patients with TSS. MNPE is a USA200 (clonal complex 30 [CC30]) S. aureus strain that produces both TSST-1 and wild-type amounts of α-toxin; most USA200 (CC30) isolates have a mutation in the α-toxin structural gene that reduces production by 50- to 100-fold (for example, strains MN8 and MNPA) (16). MNPA is a methicillin-resistant S. aureus (MRSA) strain, whereas MNPE and MN8 are methicillin sensitive.

**FIG 1:**
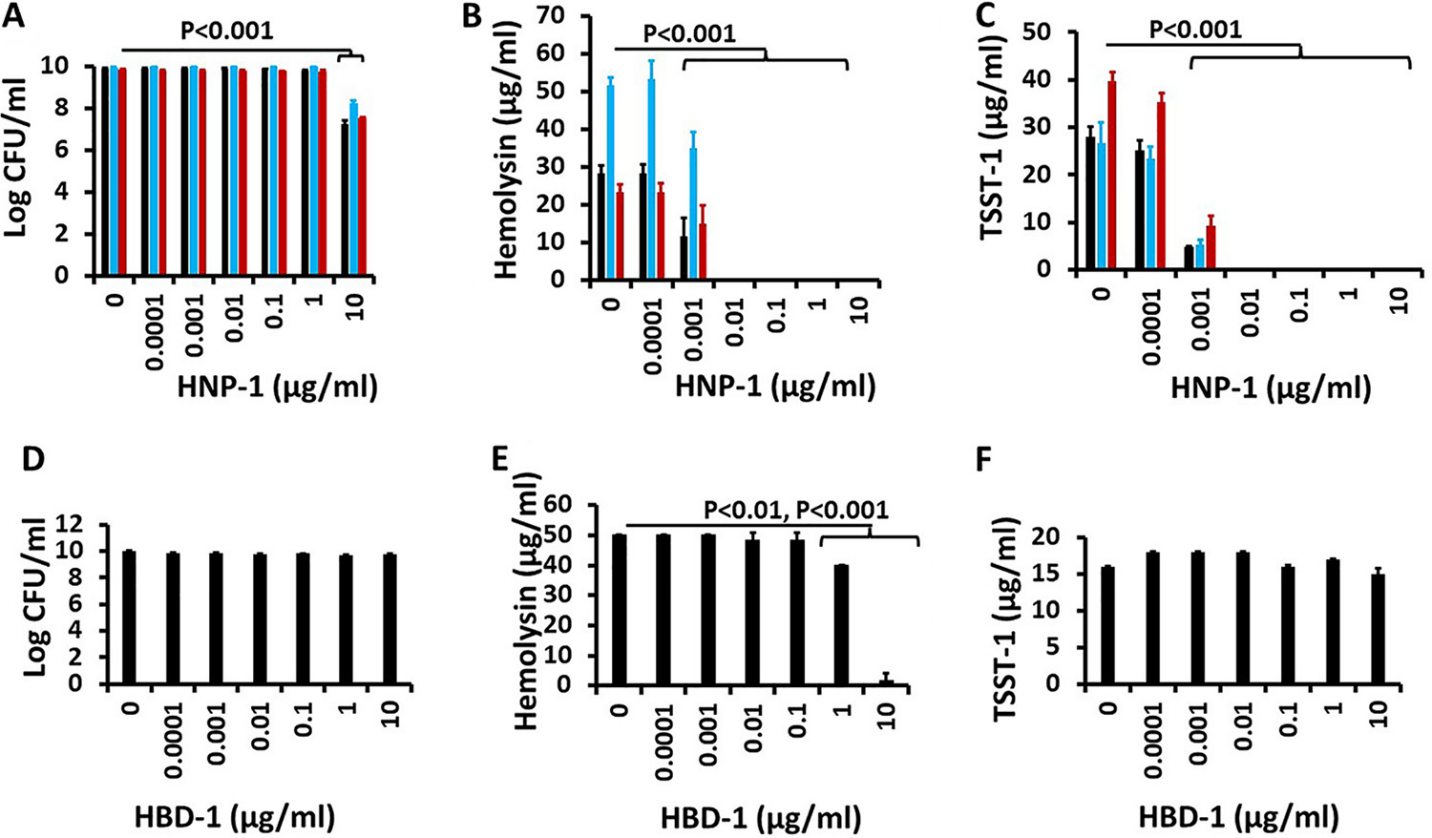
(A to F) Effect of HNP-1 and HBD-1 on growth of S. aureus (A and D) and hemolysin (B and E) and TSST-1 (C and F) exotoxin production. Black bars indicate the effect on S. aureus MNPE, blue bars indicate the effect on S. aureus MN8, and red bars indicate the effect on methicillin-resistant S. aureus (MRSA; MNPA). HNP-1 significantly inhibited the growth of all three S. aureus strains at 10 μg/mL (*P* < 0.001) (A). HBD-1 at 1 μg/mL (*P* < 0.01) and 10 μg/mL (*P* < 0.001) significantly inhibited exotoxin production (E). Hemolysins included α-, β-, γ-, δ-, ε-, and phenol-soluble modulin-α3 toxins and were measured by lysis of 5% rabbit erythrocytes.

HNP-1 significantly inhibited the growth of S. aureus MNPE, MN8, and MNPA but only at an HNP-1 concentration of 10 μg/mL (Fig. 1A). There was a 2 to 3 log reduction in 24-h growth compared to cultures without HNP-1. HNP-1 concentrations of 1 μg/mL and lower did not inhibit the growth of any of these three organisms. These observations confirm findings we have made previously regarding the relatively weak S. aureus growth-inhibitory activity of HNP-1 (17).

We next evaluated production of TSST-1 as a representative superantigen and total hemolysins (α-, β-, γ-, δ-, ε-, and phenol-soluble modulin [PSM]-α3 hemolysins, which account for greater than 99% of hemolysin activity of the three tested USA200 [clonal complex 30] strains; see Materials and Methods for explanation of total hemolysin production). We evaluated total hemolysins and the superantigen TSST-1 because total exposure of patients to these toxins is likely to be of greatest importance in disease (4). We had previously shown that all human-pathogenic strains of S. aureus produce at least one major hemolysin and at least one superantigen (4). S. aureus MNPE, MN8, and MNPA production of TSST-1 (assessed by Western blotting) and total hemolysins (assessed by bioassay) was inhibited at HNP-1 concentrations of 1 × 10^−3^ μg/mL or higher (Fig. 1B and C).

Because all three S. aureus strains responded similarly to HNP-1, only MNPE was tested with human β-defensin 1 (HBD-1). HBD-1 did not inhibit the growth of S. aureus MNPE at any HBD-1 concentration (Fig. 1D). However, this defensin inhibited hemolysin production by MNPE at HBD-1 concentrations of 1 and 10 μg/mL (Fig. 1E). HBD-1 did not inhibit TSST-1 production at any concentration.

**FIG 2:**
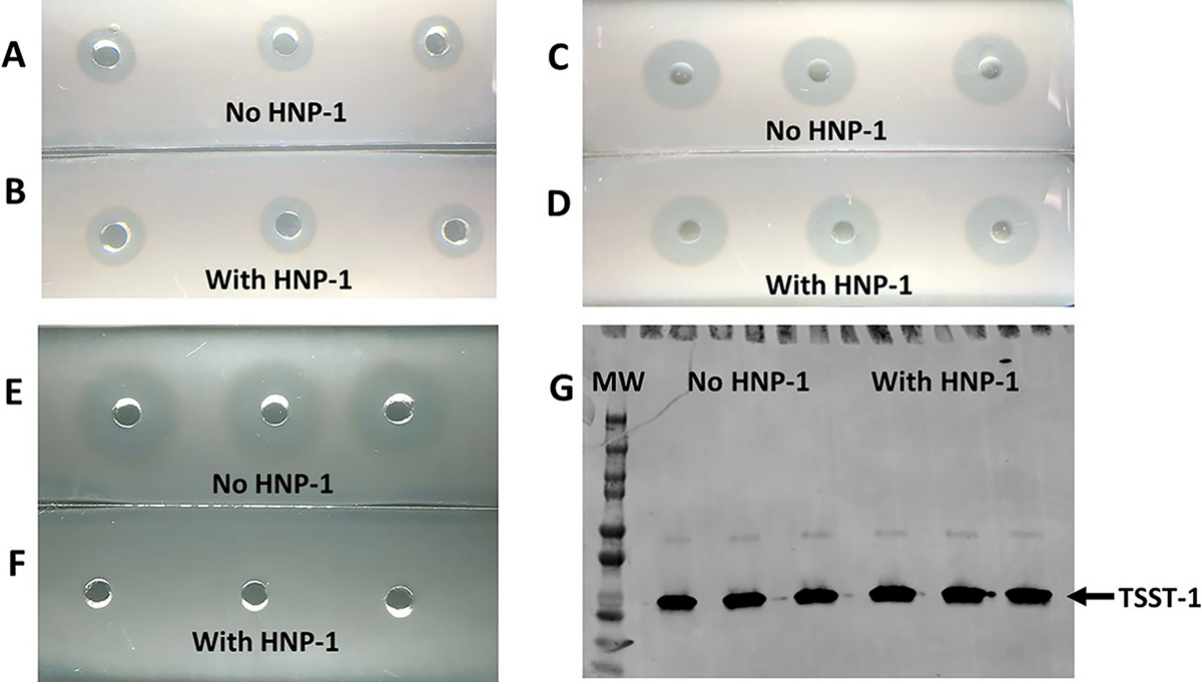
Effect of HNP-1 on detection of hemolysins by bioassay and TSST-1 by Western blotting. (A and B) S. aureus MN8 was cultured until late stationary phase at 37°C with high aeration. Sterile culture fluids (20 μL each) were incubated in triplicate with 1 μg of HNP-1 or the same volume of PBS at 37°C for 2 h. The culture fluids were then added to 0.5% (vol/vol) rabbit erythrocytes. Zones of hemolysis were measured after 18 h at 37°C. Data without HNP-1 (A) and with HNP-1 (B) are shown. (C and D) Purified α-toxin (1 μg/20 μL) was incubated in triplicate for 2 h at 37°C without HNP-1 (C) or with 1 μg HNP-1 (D). The mixtures were then added to wells in microscope slides with 0.5% rabbit erythrocytes. Zones of hemolysis were measured after 18 h at 37°C. (E and F) S. aureus MN8 was cultured until late postexponential phase without (E) or with (F) 1 μg/mL HNP-1. Sterile, cell-free filtrates were added to wells in microscope slides with 0.5% rabbit erythrocytes. Zones of hemolysis were measured after 18 h at 37°C. (G) TSST-1 (1 μg) was mixed in triplicate with water or HNP-1 for 2 h at 37°C. Then, SDS-PAGE and Western blotting with antibodies against TSST-1 were performed. Lane 1 shows the molecular weight (MW) standards, lanes 2 to 4 show TSST-1 incubated with water, and lanes 5 to 7 show TSST-1 incubated with HNP-1.

It has previously been reported that HNP-1 is able to inactivate some exotoxins, including some hemolysins and exoenzymes (18–20). This is notable for hemolysins, such as streptolysin O, that are thiol-activated hemolysins and exotoxins with enzymatic functions (18–21). Thus, it was formally possible that HNP-1 was similarly interfering with the activity or detection of staphylococcal exotoxins as opposed to preventing their production. We therefore initiated experiments to assess whether this was the case for HNP-1 effects on S. aureus exotoxins. Importantly, none of the staphylococcal hemolysins have been shown to be inactivated by oxygen, which is unlike streptolysin O. Likewise, none of the staphylococcal superantigens exhibit enzymatic activity. However, we tested both MN8 S. aureus culture fluids (after filter sterilization) of stationary-phase growth and also purified α-toxin for inactivation or hemolysin activity by HNP-1. The MN8 culture fluid should contain at least α-, β-, γ-, δ-, ε-, and PSM-α3 hemolysins. When incubated for 2 h at 37°C with HNP-1 in triplicate, there was no difference in hemolysin activity (as detected by lysis of 0.5% rabbit erythrocytes) compared to control culture fluid incubated with distilled water (Fig. 2A and B). Similarly, when HNP-1 was incubated with purified staphylococcal α-toxin (2:1 molar ratio of HNP-1:α-toxin) for 2 h at 37°C in triplicate, there was no demonstrable inactivation of α-toxin hemolytic activity (Fig. 2C and D). In contrast, when 1 μg/mL HNP-1 was added to S. aureus MN8 cultures, followed by growth into postexponential phase for 9 h, there was complete inhibition of hemolysin production compared to cultures incubated in the absence of HNP-1 (Fig. 2E and F). There was no difference in CFU/mL S. aureus (average CFU/mL was 1.1 × 10^9^/mL for strain MN8 cultured with HNP-1; average CFU/mL was 1.2× 10^9^/mL for strain MN8 cultured in the absence of HNP-1; when converted to log CFU/mL ± standard deviation, the values were 9.1 ± 0.49 for strain MN8 cultured with HNP-1 and 9.1 ± 0.15 for strain MN8 cultured in the absence of HNP-1 [these latter values were not different by Student’s *t* test analysis]).

We also incubated purified TSST-1 with HNP-1 (molar ratio 2:1 HNP-1:TSST-1) for 2 h at 37°C and examined whether the protein was stable to HNP-1, as tested by Western blotting (Fig. 2G). HNP-1 did not alter the ability to detect purified TSST-1. The data also show that our ability to detect TSST-1, whether or not the superantigen was incubated with HNP-1, is unaltered in reactivity to polyclonal antisera in Western blots.

Collectively these data indicate that the primary anti-S. aureus activity of defensin HNP-1 is inhibition of exotoxin production rather than inhibition of S. aureus growth. This was expected because the hemolysins of S. aureus are not thiol activated, and superantigens have no known enzymatic activity.

The high concentrations of HNP-1 required to inhibit S. aureus growth are likely well outside the concentrations found in tissues derived from polymorphonuclear leukocytes (PMNs) (22). However, one could argue that within neutrophils, the concentration may be sufficient to kill S. aureus. Of greater importance is the observation that HNP-1 inhibits hemolysins and the superantigen TSST-1, two major types of secreted S. aureus virulence factors required for colonization and infection by USA200 S. aureus (23, 24). This inhibitory activity is within the range of HNP-1 found in tissues. In contrast, HBD-1 did not inhibit S. aureus growth or exotoxin production at concentrations expected on human skin, a major source of HBD-1 (22). This observation may help explain why there are as many as 30 million atopic dermatitis patients in the United States infected with S. aureus (2). HBD-1, produced by keratinocytes, would not be expected to control S. aureus skin infection effectively, whereas neutrophil-derived HNP-1 would limit the inflammatory cascade resulting from S. aureus hemolysins and in so doing would likely reduce the severity of skin lesions in atopic dermatitis subjects.

**FIG 3:**
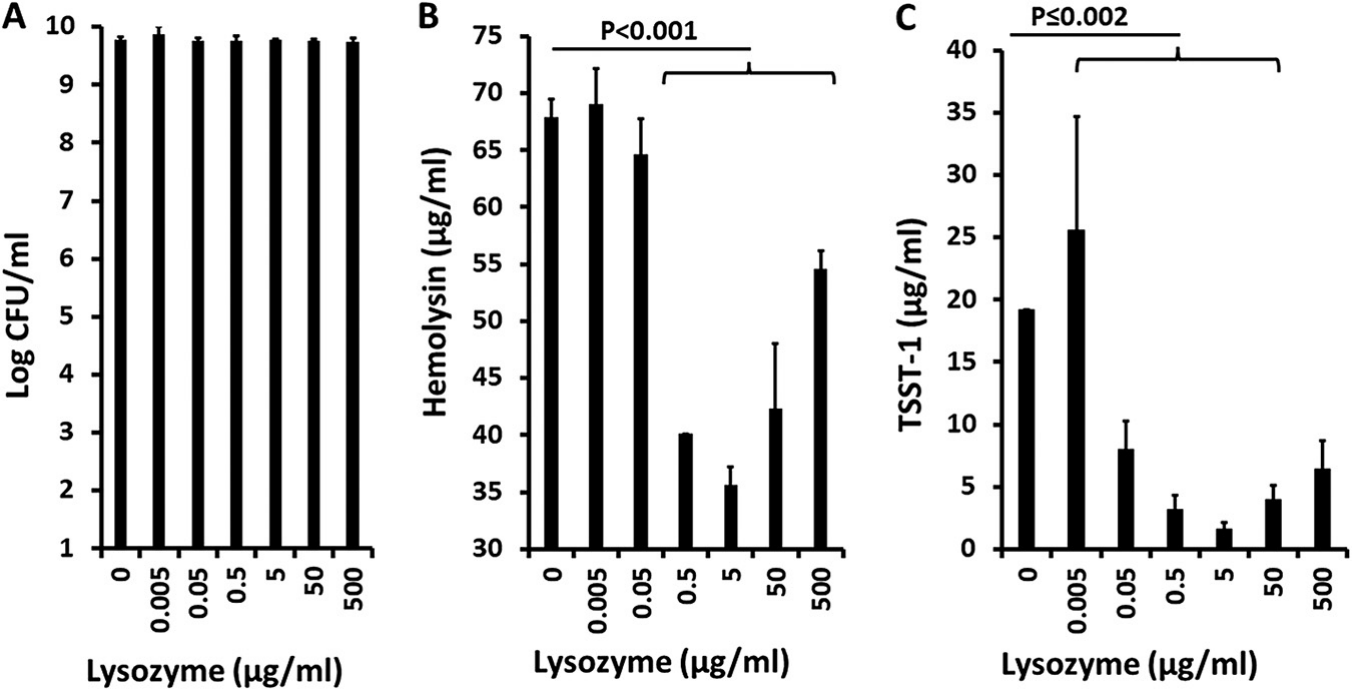
(A to C) Effect of lysozyme on growth of S. aureus MNPE (A) and on total hemolysin (B) and TSST-1 (C) production. Hemolysins include α-, β-, γ-, δ-, ε-, and phenol-soluble modulin-α3 toxins and were measured by lysis of 5% rabbit erythrocytes.

Many positively charged molecules, such as HNP-1 (17), chitosan (9), and hemoglobin peptides (17), are hypothesized to interfere with S. aureus exotoxin production in large part by their positive charges. Lysozyme is another highly positively charged (isoelectric point of ~11.3) small protein with a molecular size of approximately 15,000 Da (25). Lysozyme was first identified as an antimicrobial agent by Alexander Fleming in 1921. Since then, it has been well known that S. aureus strains are not killed by lysozyme, but the effects on exotoxin production have not been studied. We evaluated the ability of lysozyme to inhibit the growth of S. aureus MNPE and inhibit exotoxin production (Fig. 3).

As expected, lysozyme had no effect on S. aureus growth at any concentration tested (up to 500 μg/mL); the concentration of lysozyme in secretions is approximately 100 μg/mL (26). However, lysozyme significantly inhibited exotoxin production, both TSST-1 (lysozyme concentration of ≥0.05 μg/mL) and hemolysins (lysozyme concentration of ≥0.5 μg/mL). It is unclear at this time why the highest dose of lysozyme (500 μg/mL) appeared to lose some of its ability to inhibit both hemolysin and TSST-1 production. However, this may have occurred because of an overriding effect of lysozyme on another global regulator (other than SrrA/B, as studied below) required for exotoxin production and innate immune evasion, for example, S. aureus exoprotein expression R/S (SaeR/S) (27).

**FIG 4:**
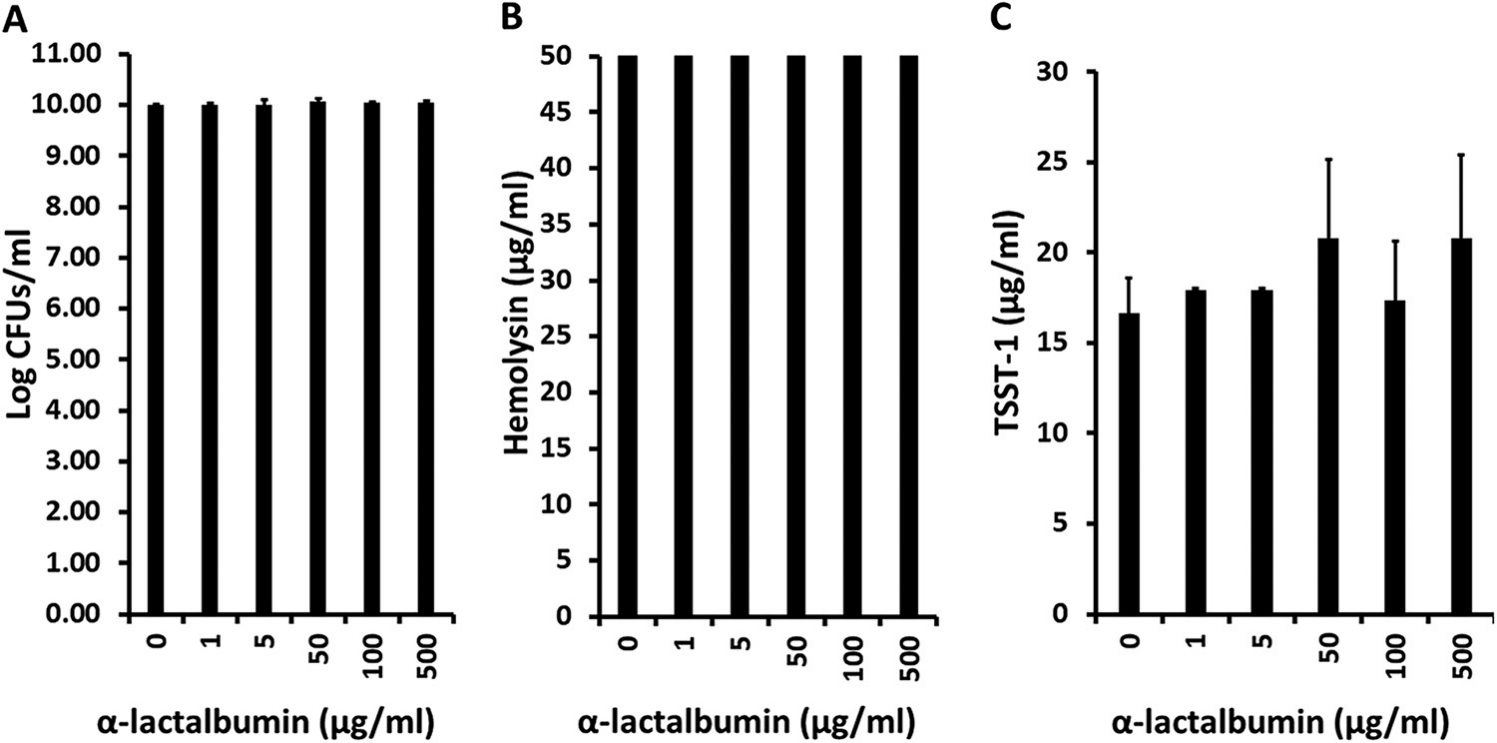
(A to C) Effect of α-lactalbumin on growth of S. aureus MNPE (A) and on total hemolysin (B) and TSST-1 (C) production. Hemolysins include α-, β-, γ-, δ-, ε-, and phenol-soluble modulin-α3 toxins and were measured by lysis of 5% rabbit erythrocytes.

>As a control experiment related to the lysozyme effect, α-lactalbumin, a molecule closely related to lysozyme in three-dimensional structure but only 40% identical in primary amino acid sequence (28), did not inhibit either the growth of S. aureus MNPE or exotoxin (TSST-1 and total hemolysin) production (Fig. 4). Overall, these data suggest that the ability of lysozyme to inhibit exotoxin production depended more on its basic isoelectric point than its three-dimensional structure. The data also suggest that the major activity of lysozyme against S. aureus *in vivo* may be to inhibit exotoxin production and not to inhibit staphylococcal growth.

We also tested whether or not HNP-1 and lysozyme inhibited exotoxin production through TCSs in our study SrrA/B. The SrrA/B two-component system (TCS) functions in S. aureus as a repressor of exotoxin production, with repression relieved by the presence of environmental oxygen (12, 13). It is well established that production of exotoxins by S. aureus is absolutely dependent on the presence of oxygen (29), making SrrA/B a critical TCS in exotoxin expression (13, 14, 30). It is also well established that the accessory gene regulatory operon (*agr*) is a quorum-sensing system with a TCS component (AgrA/C) that is also required for exotoxin production by S. aureus (31, 32). Agr is regulated by SrrA/B (13), indicating that alterations of SrrA/B would also lead to effects downstream on Agr. It is also known that molecules such as positively charged hemoglobin peptides (17), glycerol monolaurate (6), and menaquinone analogs (10) exert exotoxin-inhibitory effects through alteration of the activity of SrrA/B. These latter studies suggest that the activity of SrrA/B depends on properties in the TCR environment (hemoglobin peptides and glycerol monolaurate) or the redox potential of the S. aureus plasma membrane (menaquinone analogs) (10, 11). Importantly, it remains unknown the precise signals that are needed to derepress SrrA/B in S. aureus. Additionally, the exact earliest interaction events triggering many TCSs remain unknown.

**FIG 5:**
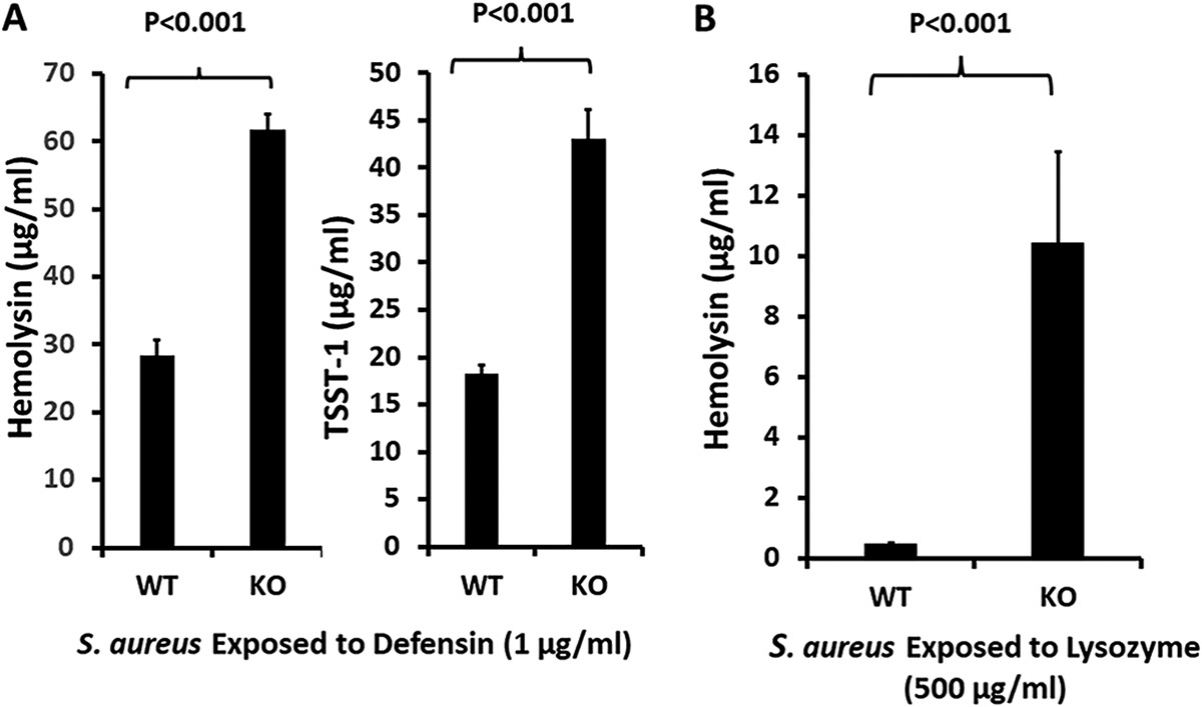
(A and B) Effect of the staphylococcal respiratory response A/B (SrrA/B) two-component system on production of hemolysins and toxic shock syndrome toxin-1 (TSST-1) in the presence of 1 μg/mL HNP-1 (A) or 500 μg/mL lysozyme (B); WT, wild-type S. aureus MN8; KO, SrrA/B clean knockout S. aureus MN8. Hemolysins include α-, β-, γ-, δ-, ε-, and phenol-soluble modulin-α3 toxins and were measured by lysis of 5% rabbit erythrocytes.

Both total hemolysins and TSST-1 were produced in larger amounts in a clean SrrA/B-knockout MN8 strain than in the wild-type MN8 strain (Fig. 5A) in the presence of HNP-1. In the presence of lysozyme (Fig. 5B), the knockout strain showed enhanced hemolysin compared to the wild-type MN8. Neither the knockout nor wild-type MN8 strain produced significant TSST-1 in the presence of lysozyme. The data strongly suggest that the ability of HNP-1 and lysozyme to inhibit exotoxin production depends on the actions of these two positively charged molecules on SrrA/B. TSST-1 and hemolysin production are controlled by additional global regulator elements, such as Agr (31, 32), Sae (27), and staphylococcal accessory regulator (Sar) (33). Differential sensitivities of the various staphylococcal global regulatory systems to inhibitory molecules may account for the apparent differential effects of HNP-1 and lysozyme on SrrA/B as determined by hemolysin and TSST-1 production. Because the precise molecular mechanisms of signaling events with SrrA/B (and indeed most TCSs) are unknown, we cannot predict how HNP-1 and lysozyme hold SrrA/B in its state of toxin repression as if the cultures were in an anaerobic environment instead of the aerobic environment used in this study.

**FIG 6:**
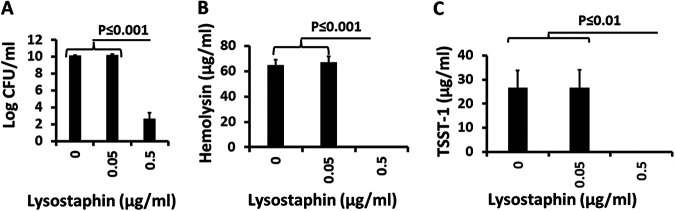
(A to C) Effect of lysostaphin on growth of S. aureus MN8 (A) and production of hemolysins (B) and TSST-1 (C) in the presence of lysostaphin (0, 0.05 μg/mL, and 0.5 μg/mL). Hemolysins include α-, β-, γ-, δ-, ε-, and phenol-soluble modulin-α3 toxins and were measured by lysis of 5% rabbit erythrocytes.

Lysostaphin is broadly used by researchers to lyse S. aureus. Lysostaphin (≥0.5 μg/mL) was highly antistaphylococcal, causing an 8-log reduction of CFU/mL compared to the untreated control (Fig. 6A), and the same concentrations inhibited exotoxin production (Fig. 6B and C). There was no lysostaphin concentration that interfered with exotoxin production while at the same time did not inhibit S. aureus growth. Thus, the lysostaphin effect on exotoxin production was linked to growth inhibition.

**FIG 7:**
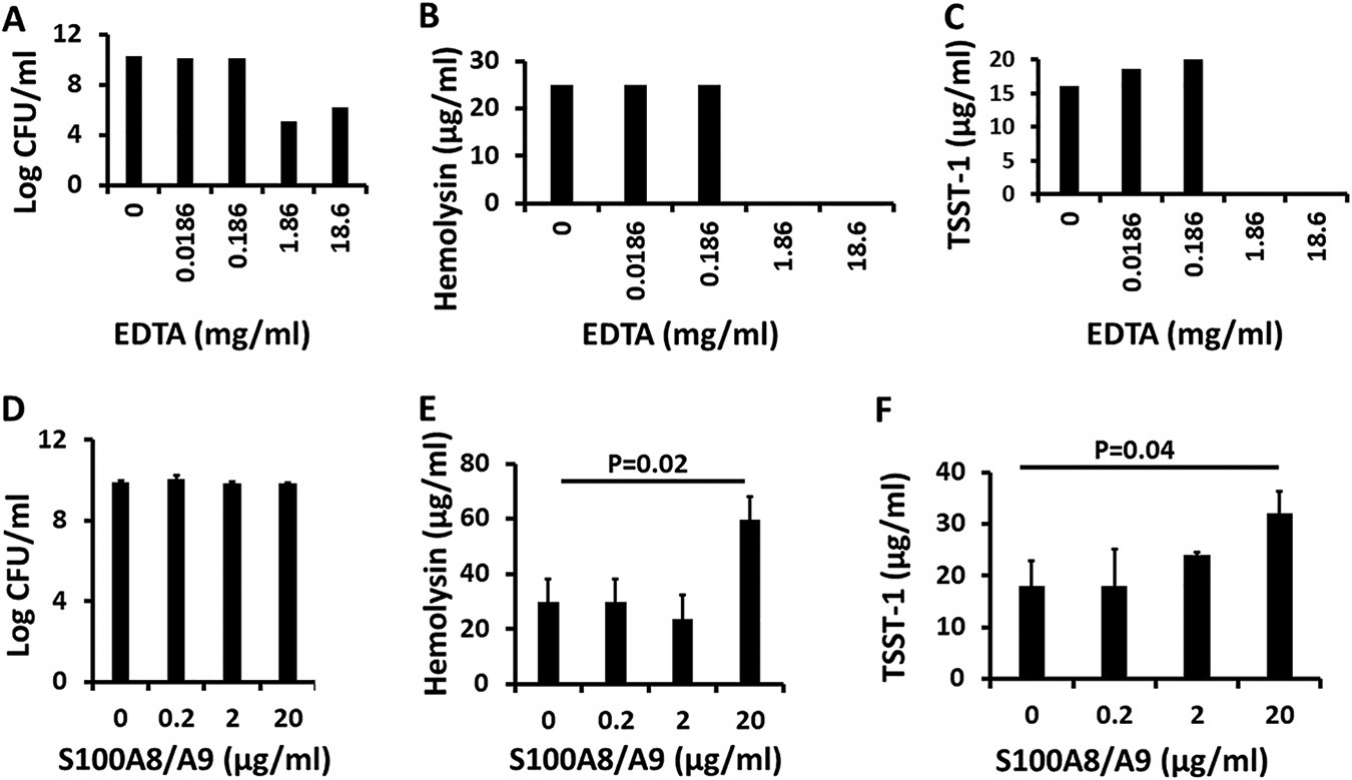
(A to F) Effect of divalent cation chelators EDTA and S100A8/A9 on growth of S. aureus MNPE (EDTA [A] and S100A8/A9 [D]) and production of hemolysins (EDTA [B] and S100A8/A9 [E]) and TSST-1 (EDTA [C] and S100A8/A9 [F]). Hemolysins include α-, β-, γ-, δ-, ε-, and phenol-soluble modulin-α3 toxins and were measured by lysis of 5% rabbit erythrocytes.

The human host also produces neutrophil and monocyte divalent cation chelators, such as the combination of S100A8/A9 (calprotectin), which functions primarily as a heterodimer to chelate calcium, attract leukocytes, and induce cytokine production (34). We compared the antistaphylococcal and exotoxin-inhibitory effect of this heterodimer to the known antistaphylococcal chelator EDTA. EDTA at 18.6 and 1.86 μg/mL caused a 4 to 5 log reduction in CFU/mL compared to untreated controls (Fig. 7A). These were the same two concentrations that inhibited TSST-1 and hemolysin production (Fig. 7B and C). Thus, the EDTA effect on exotoxin production was linked to growth inhibition. In contrast to EDTA, S100A8/A9 (added in equal amounts before the addition of S. aureus) neither inhibited S. aureus growth nor inhibited exotoxin production (Fig. 7D to F). S100A8/A9 (10 μg/mL) had no effect on growth but enhanced TSST-1 and hemolysin production. These data indicate that the S100 proteins are more likely to be proinflammatory against S. aureus as opposed to being directly antimicrobial or inhibiting exotoxin synthesis because of divalent cation chelation.

Collectively, our data show that the major activity of key antimicrobial peptides of the human innate immune system, HNP-1 and lysozyme, is to inhibit exotoxin production (both hemolysins and superantigens) rather than inhibit S. aureus growth. Both hemolysins and superantigens are required factors for S. aureus colonization and infection in humans. By inhibiting hemolysin and superantigen production, HNP-1 and lysozyme contribute to reduction of epithelial and skin infection by S. aureus. The major effects of HBD-1 and S100A8/A9 in the host may be to attract phagocytic cells to areas of S. aureus infection.

## MATERIALS AND METHODS

### Bacteria.

S. aureus strains tested belong to USA200 (clonal cluster [CC] 30) and are of low passage. The strains are stored at −80°C in the Schlievert laboratory. All strains caused TSS due to the presence of the *tstH* gene and consequent production of TSST-1. The following were the three strains used in the present study. (i) MNPE, a skin strain from a lethal case of postinfluenza TSS in a young child (35); this strain has wild-type genes for staphylococcal α-, β-, γ-, δ-, ε-, and PSM-α3 hemolysins; (ii) MN8, a menstrual vaginal TSS isolate from 1980 (36); this strain has a mutation in the α-toxin structural gene like the majority of USA200 strains, reducing hemolysin expression by 50- to 100-fold (16), but has wild-type expression of β-, γ-, δ-, ε-, and PSM-α3 hemolysins; and (iii) MNPA, a menstrual vaginal TSS isolate from a recurrent case; the strain is MRSA and has the mutation in the α-toxin structural gene but has wild-type expression of β-, γ-, δ-, ε-, and PSM-α3 hemolysins (4). All bacteria were cultured in Todd Hewitt broth (Difco, Detroit, MI) with shaking (200 rpm) at 37°C for the designated time periods. The inoculum size for each culture was approximately 5 × 10^6^ cells/mL, unless otherwise stated. This inoculum size was based on approximate vaginal cell densities in women during menstruation (37). Tests were conducted with triplicate cultures unless otherwise shown, and experiments were repeated at least two times. A clean knock out in the SrrA/B two-component system was made previously (10).

### Molecules tested.

HNP-1, lysozyme, and EDTA were purchased from Sigma-Aldrich. HBD-1 was purchased from anaSpec, Inc. (Freemont, CA). S100A8 and S100A9 were purchased from R&D Systems (Minneapolis, MN) and were mixed 50:50 before use. All molecules were dissolved in sterile distilled water for use.

### Assays for hemolysins and TSST-1.

Purified α-toxin and TSST-1 were prepared as standards for experimentation as needed. α-Toxin was purified by 80% ammonium sulfate precipitation from cultures of S. aureus MNPE (24 h of growth until well into stationary phase, with 200 rpm shaking [high aeration] at 37°C), followed by thin-layer isoelectric focusing and collection of the protein band with an isoelectric point of 7.5 (38). This band was highly lytic for rabbit erythrocytes and migrated as a single band of 33,000 Da on SDS-PAGE, the expected size of α-toxin. The toxin was also highly lethal to Dutch-belted rabbits (1.0 μg per 2 kg of body weight in <1 min) when administered intravenously.

The Schlievert laboratory also maintains highly purified β-toxin (39), δ-toxin (synthesized), bicomponent γ-toxins (40), ɛ-toxin (41), and PSM-α3 toxin (synthesized). All of these hemolysins have been determined in the Schlievert laboratory to be lytic to rabbit erythrocytes, validating the use of α-toxin as the experimental standard for determination of total hemolysin production as necessary.

**FIG 8:**
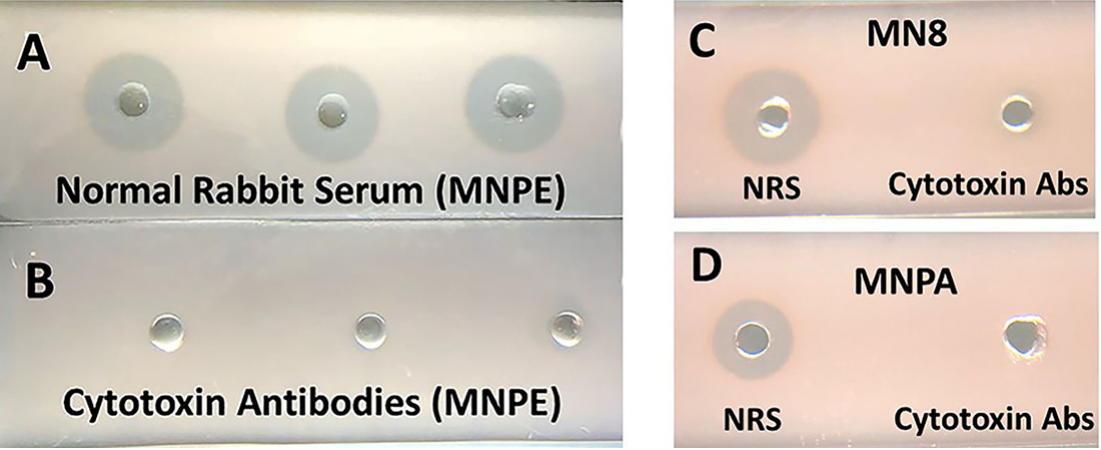
(A and B) Inhibition of hemolysin activity of sterile triplicate MNPE culture fluids (12 μL) after incubation for 2 h at 37°C with collectively 2-μL amounts of rabbit antibodies against α-, β-, γ-, δ-, ε-, and PSM-α3 cytotoxins (2 μL each, 12 μL total) or 12 μL of normal rabbit serum. The culture fluids were subsequently added to wells in microscope slides containing 0.5% rabbit erythrocytes and 0.85% agarose in PBS. After 18 h of incubation at 37°C, the zones of lysis were determined. Data with normal rabbit serum (A) and with antibodies to the 6 hemolysins (B) are shown. (C and D) Inhibition of hemolysin activity of sterile culture fluids of MN8 (C) or MNPA (D) after incubation for 2 h at 37°C with collectively 2-μL amounts of rabbit antibodies against α-, β-, γ-, δ-, ε-, and PSM-α3 cytotoxins (2 μL each, 12 μL total) or 12 μL of normal rabbit serum (NRS). The sterile culture fluids were subsequently added to wells in microscope slides containing 0.5% rabbit erythrocytes and 0.85% agarose in PBS. After 18 h of incubation at 37°C, the zones of lysis were determined; Abs, antibodies.

The combinations of α-, β-, γ-, δ-, ε-, and PSM-α3 toxins account for greater than 99% of the hemolysin activity produced by the three USA200 strains tested in our current studies. This was determined through neutralization studies with specific, hyperimmune rabbit antisera to each of the purified hemolysins (Fig. 8A to D). Our experience is that nearly 100% of USA200 (CC30) strains of S. aureus have the wild-type β-toxin gene. In many other S. aureus clonal clusters, the β-toxin structural gene is inactivated by bacteriophage insertion, at least as tested *in vitro* (42). β-Toxin is well-known as the hot-cold hemolysin for sheep erythrocytes. However, the toxin is not a hot-cold hemolysin for rabbit erythrocytes. Instead, it causes direct hemolysis at 37°C like other staphylococcal hemolysins. δ-Toxin belongs to the same family of hemolysins as PSM-α3. These are the two dominant small-peptide hemolysins (20 to 26 amino acids) of our USA200 (CC30) strains. γ-Toxins are octamer bicomponent hemolysins (AB and BC components). All USA200 (CC30) strains we have tested produce γ-toxins, which are lytic to rabbit erythrocytes. Finally, all S. aureus strains produce the recently described ε-hemolysin, which again is lytic to rabbit erythrocytes.

The assay for total hemolysin (α-, β-, γ-, δ-, ε-, and PSM-α3 toxins) production by S. aureus strains was lysis of 5% rabbit erythrocytes, with α-toxin lysis as the standard. The diameter of lysis squared was proportional to the hemolysin concentration. Culture fluids were added to 5% rabbit erythrocytes (vol/vol) in agar in phosphate-buffered saline (PBS; 0.005 M NaPO_4_ and 0.15 M NaCl [pH 7.2]) in 4-mm wells, punched in petri plates, containing 20 mL of the erythrocyte-agarose mixture; cells were incubated for 24 h at 37°C in the presence of 5% CO_2_. Subsequently, the diameters of lysis of culture fluids, and when necessary were compared to α-toxin standards.

In some experiments (shown in Fig. 2 and 8), total hemolysin was measured as lysis of rabbit erythrocytes (0.5% [vol/vol] in agarose in PBS in 4-mm wells, punched in microscope slides, containing 4 mL of the erythrocyte-agarose mixture, followed by incubation overnight at 37°C in the presence of 5% CO_2_).

The Schlievert laboratory is the original source of TSST-1 and antibodies used as standards (29, 43, 44). TSST-1 was purified in the same way as α-toxin, except the source strain was S. aureus RN4220 with cloned TSST-1 on plasmid pCE107 (39), and the superantigen was collected after 80% absolute ethanol precipitation from cultures followed by thin-layer isoelectric focusing (38). The purified TSST-1 migrated as a single band on SDS-PAGE (38). Rabbits were hyperimmunized against TSST-1 by subcutaneous injection (25 μg/injection) of TSST-1 emulsified in Freund’s incomplete adjuvant until the sera gave an enzyme-linked immunosorbent assay (ELISA) titer of approximately 100,000. TSST-1 was assayed by Western blotting (45) and quantitative double immunodiffusion (5).

In one set of experiments, triplicate aliquots of purified α-toxin (1 μg/20 μL) or TSST-1 (1.0 μg/20 μL) were incubated at 37°C for 2 h with 1 μg/20 μL HNP-1. Subsequently, the samples were either tested for lysis on rabbit erythrocytes on microscope slides or were subjected to SDS-PAGE and Western blotting (46).

### Statistics.

Means ± standard deviation values were determined. Student’s *t* tests of unpaired data were used to determine differences in means.
